# Effects of High-Intensity Interval Training on Blood Pressure Levels in Hypertensive Patients: A Systematic Review and Meta-Analysis of Randomized Clinical Trials

**DOI:** 10.3390/life14121661

**Published:** 2024-12-14

**Authors:** Luis Romero-Vera, David Ulloa-Díaz, Sergio Araya-Sierralta, Francisco Guede-Rojas, Oscar Andrades-Ramírez, Claudio Carvajal-Parodi, Gustavo Muñoz-Bustos, María Matamala-Aguilera, Darío Martínez-García

**Affiliations:** 1 Facultad de Medicina y Ciencias de la Salud, Escuela de Kinesiología, Magíster en Fisiología Clínica del Ejercicio, Universidad Mayor, Santiago 8580745, Chile; lromero01@mayor.cl; 2 Department of Sports Sciences and Physical Conditioning, Universidad Católica de la Santísima, Concepción 4030000, Chile; 3 Facultad de Humanidades y Educación, Universidad de Atacama, Copiapó 1531772, Chile; sergio.araya@uda.cl; 4 Exercise and Rehabilitation Sciences Institute, School of Physical Therapy, Faculty of Rehabilitation Sciences, Universidad Andres Bello, Santiago 7591538, Chile; francisco.guede@unab.cl; 5 Facultad de Salud y Ciencias Sociales, Escuela de Ciencias de la Actividad Física, Universidad de las Américas, Concepción 4030000, Chile; oandrades@udla.cl; 6 Facultad de Odontología y Ciencias de la Rehabilitación, Escuela de Kinesiología, Universidad San Sebastián, Concepción 4081339, Chile; claudio.carvajal@uss.cl; 7 Facultad de Salud, Escuela de Nutrición y Dietética, Universidad Santo Tomás, Iquique 1100000, Chile; g.munoz.bustos@gmail.com; 8 Independent Researcher, Concepción 4030000, Chile; mjmatamalag@gmail.com; 9 Department of Physical Education and Sport, Faculty of Sport Sciences, University of Granada, 18011 Granada, Spain; dariomg@ugr.es

**Keywords:** high blood pressure, blood pressure, high-intensity interval training, intensity, control, manipulation

## Abstract

Objective: This systematic review and meta-analysis aimed to (I) evaluate the evidence on the effects of high-intensity interval training (HIIT) on systolic blood pressure (SBP) and diastolic blood pressure (DBP) in hypertensive patients; (II) determine whether HIIT impacts SBP and DBP differently; and (III) assess the clinical relevance of these effects. Methods: A comprehensive search was conducted across multiple electronic databases, resulting in the inclusion of seven randomized clinical trials in the meta-analysis. The outcomes were analyzed using random-effects models to compute mean differences (MD) and standardized mean differences (SMD) for SBP and DBP. Results: A small reduction in SBP was observed with HIIT interventions (MD −3.00; 95% CI −4.61 to −1.39; *p* < 0.0001; SMD −0.28; 95% CI −0.42 to −0.13; *p* = 0.0003). However, no statistically significant reductions were detected for DBP (MD −0.70; 95% CI −1.80 to 0.39; *p* = 0.21; SMD −0.07; 95% CI −0.22 to 0.08; *p* = 0.35). Despite demonstrating statistical significance for SBP, the effects did not reach clinical relevance. Conclusions: HIIT interventions yield small reductions in SBP, with minimal impact on DBP. These findings suggest limited clinical relevance in the management of hypertension. Further randomized controlled trials are necessary to standardize HIIT protocols, with specific emphasis on intensity control and manipulation, to better understand their potential role in hypertensive populations.

## 1. Introduction

Hypertension (HTN) is among the leading causes of morbidity and mortality worldwide. It is projected that by 2025, over 1.5 billion individuals will be diagnosed with HTN [1], making it one of the most critical health risks associated with chronic diseases [2,3]. There is consensus in the scientific community that increasing physical activity levels and engaging in structured exercise programmes significantly reduces the risk of developing HTN [4,5]. Current evidence highlights that dietary control, sodium intake reduction, and regular physical exercise are some of the most promising non-pharmacological strategies for the prevention and treatment of HTN [6].

While low- to moderate-intensity continuous exercise has the strongest evidence base for its effectiveness [7,8], research exploring alternative exercise modalities, including those with moderate-to-high intensity, is growing [9,10]. In particular, high-intensity interval training (HIIT) has gained attention as a feasible and time-efficient option for clinical interventions in individuals with HTN [11,12]. The appeal of HIIT lies in its shorter session durations, which enhance adherence to exercise programmes [13]. Studies conducted on athletes and the general population have demonstrated that HIIT positively influences physical performance [14], body weight management, lipid profiles [15], maximal and peak oxygen uptake (VO_2max_ and VO_2peak_), anaerobic threshold [16], and ventilatory threshold displacement [17,18,19]. It has been demonstrated that HIIT offers benefits for endothelial function, reduces peripheral vascular resistance, and improves control of adrenal sympathetic activity—factors that may enhance the regulation of blood pressure control mechanisms [20,21].

The effects of HIIT on systolic blood pressure (SBP) [22,23,24] and diastolic blood pressure (DBP) [25,26] have been documented in various studies. However, when compared to other exercise modalities, HIIT demonstrates comparatively smaller effects on SBP and only slightly outperforms walking interventions in reducing DBP [27]. A recent meta-analysis by Edward et al. [27] compared HIIT to other exercise forms in normotensive, pre-hypertensive, and hypertensive individuals, revealing significant reductions in resting SBP and DBP across all exercise modalities, except for aerobic interval training (AIT), the term used for HIIT in this context. Intervention strategies for individuals with hypertension include both pharmacological and non-pharmacological treatments. Non-pharmacological treatments such as exercise can significantly improve SBP and DBP in middle-aged and older adults, contributing to enhanced physical and mental health [28,29].

Although HIIT has been shown to reduce SBP and DBP, its efficacy remains unclear when compared to pharmacological treatments. For instance, the average reductions achieved through monotherapy with ACE inhibitors are approximately −8.5 mmHg for SBP and −4.7 mmHg for DBP [30,31,32,33], while thiazide diuretics produce reductions of −8.8 mmHg and −4.4 mmHg, respectively [34,35,36,37,38]. Calcium channel blockers (CCB) [39,40,41,42,43], β-blockers [44,45,46], and angiotensin II receptor antagonists [47] yield even greater reductions. Moreover, combined pharmacological therapies achieve reductions of up to −19.9 mmHg for SBP and −10.7 mmHg for DBP when using three medications [47,48]. Recently, Laurent [49] described that monotherapy or pharmacological combinations present adverse effects such as increased glucose intolerance, hypoglycemic symptoms, hyponatremia (depletion and dilution of Na^+^), hypokalemia, metabolic alkalosis, hypovolemia, hypotension, and, to a lesser extent, hyperuricemia, hypocalcemia, hypomagnesemia, hyperglycemia, hyperlipidemia, urinary urgency, and sexual dysfunction. Among non-pharmacological strategies, sodium reduction results in an average decrease of −6.81 mmHg in SBP and −3.85 mmHg in DBP [50]. In contrast, the impact of HIIT on hypertensive levels has not been well established.

Systematic reviews and meta-analyses indicate that HIIT can reduce SBP and DBP [51,52], particularly when comparing HIIT intervention groups with control groups. However, disparities in the design, control, and manipulation of HIIT protocols often limit the reproducibility of these findings and might explain the inconsistent results observed across studies. Furthermore, it remains uncertain whether these effects are sufficient to induce clinically meaningful changes in SBP and DBP in hypertensive individuals. Existing meta-analyses predominantly report mean differences (MD), without employing standardized mean differences (SMD) as a marker of the relative variation in blood pressure, potentially biasing the analysis of HIIT’s effects and their clinical relevance.

Therefore, the present study has the following aims: (I) evaluate the level of evidence on the effects of HIIT interventions on SBP and DBP; (II) determine whether HIIT impacts SBP and DBP differently; and (III) assess the clinical relevance of these effects in hypertensive patients.

## 2. Methods

### 2.1. Study Design

**Figure 1:**
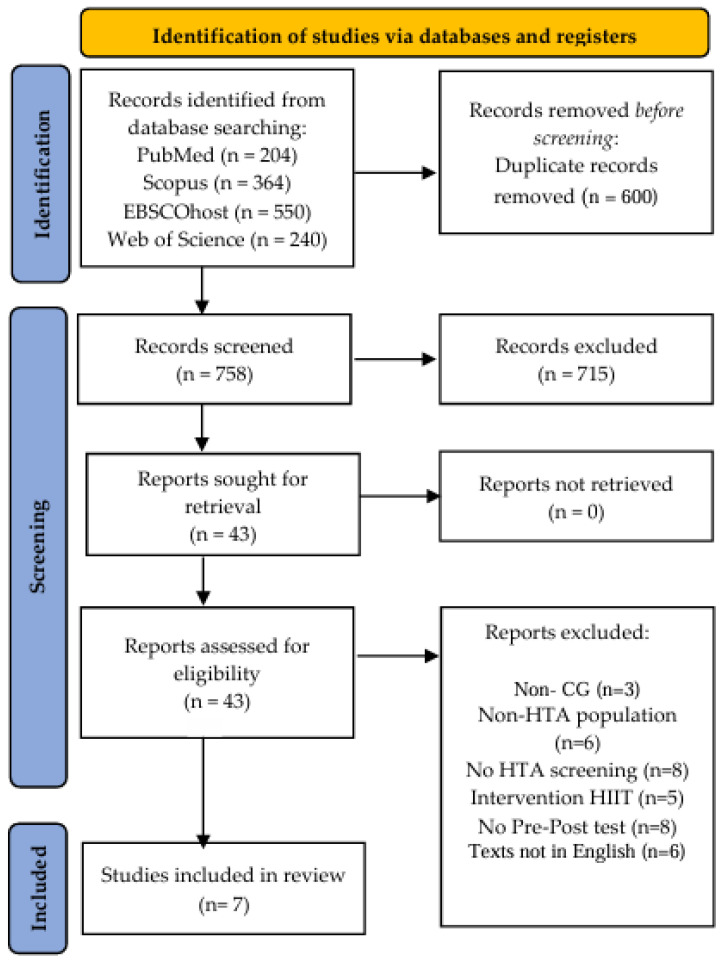
Preferred Reporting Items for Systematic Reviews and Meta-Analyses flow diagram, showing article selection process.

This systematic review and meta-analysis was conducted following the 2020 PRISMA (Preferred Reporting Items for Systematic Reviews and Meta-Analyses) guidelines [53]. The study protocol was registered in the INPLASY database under reference number 202480131 (Figure 1).

### 2.2. Search Strategy

Systematic searches were performed across four electronic databases: Scopus, PubMed, EBSCOhost, and Web of Science, covering all publications up to 1 April 2024. The search strategy combined the following keywords:

Hypertension-related terms: “Hypertension”, “High Blood Pressure”, “Arterial Hypertension”, “Primary Hypertension”, and “Blood Pressure, High”.

HIIT-related terms: “High-Intensity Interval Training”, “Interval Training”, “High-Intensity Interval”, “High-Intensity Intermittent Exercise”, and “HIIT”.

Boolean operators (AND/OR) were used to refine the search. The complete search strategy for PubMed is provided in Table 1. Duplicate records were removed, and two independent reviewers (D.U. and F.G.) screened titles, abstracts, and full texts of English-language articles.

### 2.3. Eligibility Criteria

Studies were included if they met the following eligibility criteria: (i) hypertensive adults (≥18 years), as defined by European Society of Hypertension/European Society of Cardiology (ESC/ESH) guidelines [54], including pre-hypertension (SBP/DBP = 130–139/85–89 mmHg) and hypertension (SBP/DBP ≥ 140/90 mmHg), (ii) HIIT conducted using a treadmill or cycle ergometer, (iii) studies comparing HIIT with active recovery to another exercise modality or a non-exercise control group, (iv) changes in SBP and DBP pre- and post-intervention, and (v) randomized clinical trials (RCTs).

Studies were excluded if (i) the HIIT protocol included sprint interval training (SIT), high-intensity intermittent exercise (HIIE), or aerobic interval training (AIT), (ii) subjects were athletes, young adults, or from a non-hypertensive population, (iii) the study involved a single HIIT session or lacked pre- and post-intervention blood pressure measurements, or (iv) the study was not published in English.

### 2.4. Data Extraction

Two independent reviewers extracted the following information from eligible studies: (i) study characteristics (title, authors, and publication year), (ii) intervention details (HIIT type, session duration, frequency, and volume), (iii) intensity control methods (e.g., %HR_max_, %HRR, %VO_2peak_, or self-selected intensity [SSI]), (iv) pre- and post-intervention SBP and DBP measurements, and (v) pharmacological interventions, if any, and where effect sizes were not reported, they were calculated using available data.

### 2.5. Risk of Bias Assessment

The risk of bias for each study was assessed independently by two reviewers (G.M. and F.G.) using the Cochrane^®^ Risk of Bias 2 (RoB2) tool (Cochrane collaboration, Oxford, UK) [55]. Domains evaluated included random sequence generation, allocation concealment, participant and personnel blinding, outcome assessment blinding, incomplete outcome data, and selective reporting. Discrepancies were resolved by consensus or consultation with a third reviewer (O.A.).

### 2.6. Publication Bias

Publication bias was examined visually using Begg’s funnel plot and statistically using Begg’s rank correlation test [56] and Egger’s regression test [57]. The Duval and Tweedie “trim-and-fill” method [58] was applied to account for potential bias. Sensitivity analyses were performed to determine the robustness of the results.

### 2.7. Statistical Analyses

All calculations were conducted using a Microsoft Excel (Microsoft, Redmond, WA, USA) spreadsheet containing data extracted from each publication. Review Manager (RevMan) version 5.4.5 was used for all the statistical analyses’ forest plots. The Cochrane Q statistic [59] was used to assess heterogeneity between studies. Heterogeneity is a measure of the differences in main effects between studies. Additionally, I^2^ statistics were used to evaluate heterogeneity (I^2^ > 50%).

The effects of HIIT programs on SBP and DBP were calculated for each included study, following coding of the differences between experimental and control groups and their standard deviations (SDs). The mean difference (MD) and standardized mean difference (SMD) were calculated by subtracting the post-intervention values of BP measures in every group. Data were required to take these forms: (a) the mean and SDs (pre- and post-intervention); (b) 95% confidence interval (CI) data for pre- to post-intervention changes for each group; or when this was unavailable, (c) actual *p*-values for pre- to post-intervention changes for each group; or, if only the level of statistical significance was available, (d) default *p*-values (e.g., *p* < 0.05 becomes *p* = 0.49, *p* < 0.01 becomes *p* = 0.0099, and *p* when not significant becomes *p* > 0.05). The random effects inverse variance (IV) was used with the measurement of the effect of SMD. The analysis of ES was conducted with a random effects model estimated using the DerSimonian and Laird method [60]. A random effects model was incorporated when the assumption was that the data demonstrated effects across studies that were randomly situated around a central value. Forest plots were generated to demonstrate the differences in the experimental intervention effects on blood pressure variables and ESs within the respective 95% CIs. Combining estimates then allowed for the assessment of a pooled effect. The reciprocal sums of two variances were accounted for, including the estimated variance associated with the study and the estimated variance component due to the variation between studies. A sensitivity analysis was conducted to identify highly influential studies that might have biased the analysis.

The study-specific weight was derived as the inverse of the square of the respective standard errors. ESs of <0.2, <0.5, <0.8, and >0.8 were considered trivial, small, moderate, and large, respectively [61].

## 3. Results

### 3.1. Study Selection

The database search yielded 1358 publications. After the removal of 600 duplicates, 758 unique records remained. Title and abstract screening excluded 715 articles due to irrelevance. A total of 43 full-text articles were evaluated for eligibility, of which 36 were excluded for the following reasons: absence of a control group (*n* = 3); non-hypertensive populations (*n* = 6); lack of hypertension-specific screening (*n* = 8); interventions involving a single HIIT session or alternative exercise protocols (*n* = 5); absence of pre- and post-intervention blood pressure measurements (*n* = 8); and non-English-language publications (*n* = 6). Seven randomized clinical trials were included in the final meta-analysis [62,63,64,65,66,67,68] (Figure 1).

### 3.2. Study Characteristics

The included studies, published between 2018 and 2024, represent diverse regions, including South America, North America, Europe, and Asia. Two studies included only female participants [64,67], one study included exclusively male participants [66], while the remaining four studies featured mixed-gender cohorts [62,63,65,68]. Collectively, the studies analyzed a total of 573 participants, comprising 375 hypertensive individuals who underwent HIIT interventions and 198 hypertensive or normotensive participants serving as controls. Participant ages ranged from 40 to 65 years. Detailed characteristics of the included studies are presented in Table 2.

### 3.3. Assessment of Bias

**Figure 2:**
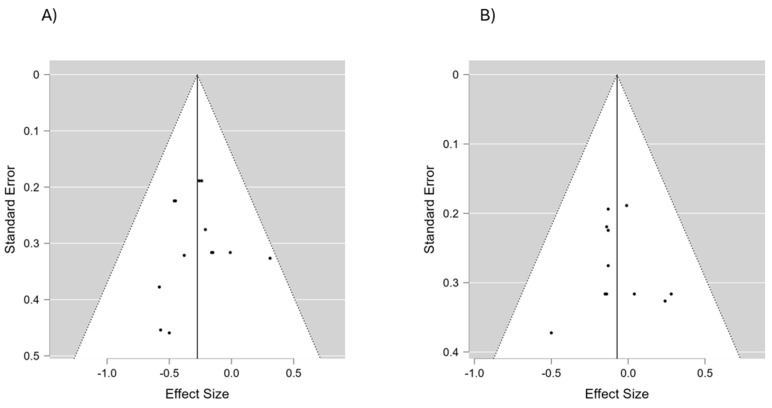
A funnel plot of the included studies to assess the potential risk of bias in both SBP (**A**) and DBP (**B**).

The authors did not detect any publication bias or heterogeneity (I2 = 0% in all cases) in this meta-analysis. The funnel plot reveals that most data points within the plot are within the funnel, indicating that bias and between-study heterogeneity did not exist in the studies. If bias did exist, the data points would have produced results outside of the reverse funnel, denoting asymmetry and bias (Figure 2).

### 3.4. Effects of HIIT on Systolic Blood Pressure

**Figure 3:**
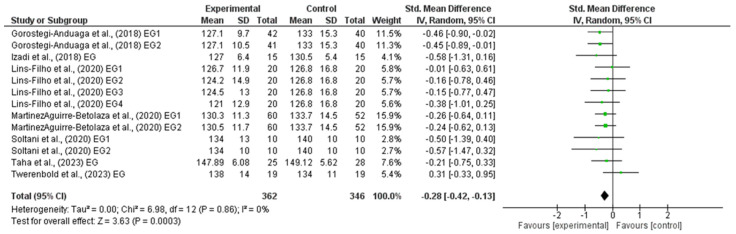
Forest plot detailing standardized mean difference and 95% confidence intervals (CI) for the effect of high-intensity interval training on SBP.

The outcomes for SBP are shown in the forest plot in Figure 3. The difference in SBP between the experimental and control group measurements was assessed via a meta-analysis of all the included studies. Due to inherent human variability, a random effects model was incorporated with I2 and used to assess blood pressure measures. There was no heterogeneity detected in all seven studies included in the meta-analysis (I2 = 0%). A small effect was observed when a random effects analysis was applied for SBP outcomes (MD −3.00; 95% CI −4.61; −1.39; *p* < 0.0001; and SMD −0.28; 95% CI −0.42; −0.13; *p* = 0.0003).

### 3.5. Effects of HIIT on Diastolic Blood Pressure

**Figure 4:**
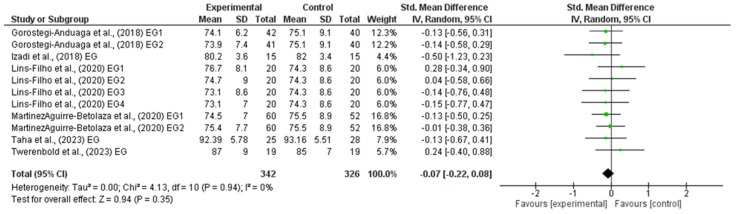
Forest plot detailing standardized mean difference and 95% confidence intervals (CI) for the effect of high-intensity interval training on DBP.

The outcomes for DBP are shown in the forest plot in Figure 4. The difference in DBP between experimental and control group measurements was assessed via a meta-analysis of all the included studies. Due to inherent human variability, a random effects model was incorporated with I2 and used to assess blood pressure measures. There was no heterogeneity detected in all seven studies included in the meta-analysis (I2 = 0%). A small effect was observed when a random effects analysis was applied for DBP outcomes (MD −0.70; 95% CI −1.80; 0.39; *p* = 0.21) and (SMD −0.07; 95% CI −0.22; 0.08; *p* = 0.35).

### 3.6. Quality Assessment

**Figure 5:**
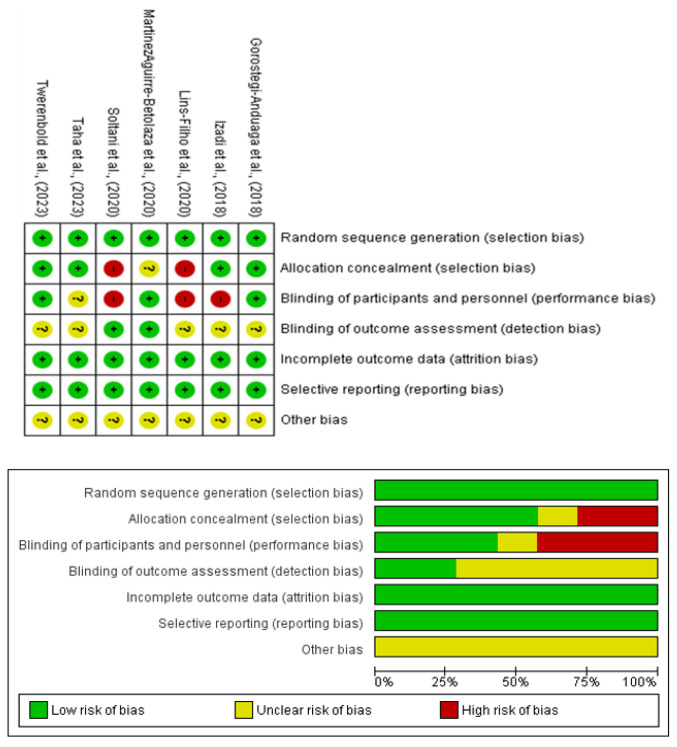
Risk-of-bias assessment of randomized clinical trials using the Cochrane evaluation tool.

Most studies provided sufficient information to assess the risk of bias across domains, such as random sequence generation, allocation concealment, participant and personnel blinding, and outcome assessment (Figure 5). The funnel plot (Figure 2) revealed no asymmetry, indicating minimal publication bias. Additionally, statistical tests confirmed the absence of bias, with no significant heterogeneity detected across the studies.

## 4. Discussion

The primary objectives of this study were to (I) evaluate the evidence on the effects of high-intensity interval training (HIIT) interventions on systolic blood pressure (SBP) and diastolic blood pressure (DBP); (II) determine whether these effects differ between SBP and DBP; and (III) assess the clinical relevance of these effects in hypertensive populations. The findings demonstrated a statistically significant but modest reduction in SBP (SMD −0.28; 95% CI −0.42 to −0.13; *p* = 0.0003) following HIIT interventions. However, the observed reduction did not reach clinical significance (MD −3.00; 95% CI −4.61 to −1.39; *p* < 0.0001). For DBP, no significant reductions were detected (MD −0.70; 95% CI −1.80 to 0.39; *p* = 0.21). These findings support the hypothesis that HIIT produces differential effects on SBP and DBP, with a limited overall impact on hypertension management.

Post-exercise reductions in blood pressure are primarily mediated by neural and hemodynamic mechanisms. These neurocirculatory control mechanisms reduce sympathetic activity and improve baroreflex function [69,70,71]. Additionally, increased nitric oxide (NO) bioavailability [69,72], improved redox state [73,74], and enhanced peripheral vascular endothelial function [69,70,71,72,73,74] contribute to hypertension control, although the mechanisms underlying the antihypertensive effects of exercise remain insufficiently understood [69,70,71,72,73,74].

Long-term adaptations include reduced sympathetic activity and increased vagal tone [7]. Our findings align with previous meta-analyses indicating HIIT’s effectiveness in reducing SBP [75,76,77]. For instance, Leal et al. [78] reported a mean SBP reduction of −5.64 mmHg (95% CI −9.52 to −1.69; *p* = 0.005), while Li et al. [52] demonstrated reductions of −4.14 mmHg (95% CI −6.98 to −1.30; *p* < 0.001) in hypertensive populations. However, our study observed smaller reductions, likely due to differences in inclusion criteria and variations in HIIT protocol designs. Discrepancies in intensity control and session structure may have contributed to the variability in the reported effects.

The limited reduction in DBP observed in this study mirrors findings from other investigations [79,80]. These differences may reflect the distinct physiological responses of SBP and DBP to exercise stimuli [75,76,77]. Other studies have highlighted that HIIT tends to have a smaller impact on DBP compared to other exercise modalities, such as isometric training [27]. This reinforces the notion that while HIIT provides cardiovascular benefits, its effects on DBP may be less pronounced.

The modest SBP reductions achieved through HIIT pale in comparison to pharmacological treatments, which typically reduce SBP by 8.5–10.3 mmHg and DBP by 4.4–6.7 mmHg depending on the drug class [30,31,32,33,34,35,36,37,38,39,40,41,42,43,44,45,46,47,48]. Even non-pharmacological interventions, such as sodium reduction, achieve average reductions of −6.81 mmHg for SBP and −3.85 mmHg for DBP [50]. These comparisons underscore the limited standalone efficacy of HIIT in controlling hypertension. However, its time-efficient nature may support adherence to exercise programmes, especially in clinical populations where sustained engagement is critical.

HIIT as a training methodology has been associated with high levels of adherence to intervention programs [13,14]. However, there is still no consensus on the risks or safety issues related to controlling intensity variables, according to HIIT design recommendations, a matter that remains unresolved within the scientific community [13,14,17]. When compared to other exercise modalities, such as moderate-intensity continuous training (MICT) or isometric exercise, HIIT shows similar or smaller effects on SBP and DBP [27,81]. For instance, Edwards et al. [27] reported reductions of −4.08 mmHg for SBP with HIIT compared to −4.49 mmHg for traditional aerobic training and −8.24 mmHg for isometric exercise. These findings suggest that while HIIT may offer a feasible option for individuals with time constraints, alternative exercise modalities could be more effective in reducing blood pressure.

The variability in HIIT protocols, particularly in intensity control, session duration, and frequency, presents significant challenges to standardization and reproducibility.

To ensure individualization in HIIT prescription and the optimal training zone, continuous monitoring and control of intensity are required throughout the training period [13,14,82]. Therefore, selecting HIIT as a training method demands greater control over training variables when designing interventions aimed at reducing blood pressure levels in hypertensive patients [82,83,84]. Most included studies relied on measures such as %HR_max_, %HRR, or %VO_2peak_ to control intensity [62,63,64,65,66,67,68], but inconsistencies in these parameters likely influenced the outcomes. For example, studies using self-selected intensity (SSI) often failed to achieve optimal exertion levels [64,68], leading to trivial effects on SBP and DBP. Additionally, medications such as β-blockers may alter physiological responses to HIIT, further complicating intensity prescription [44]. Future research should focus on standardizing HIIT protocols to optimize intensity manipulation and ensure adequate progression throughout the intervention. Parameters such as %HR_max_, %VO_2peak_, and SSI should be rigorously controlled and adjusted to match individual capabilities. Investigating the effects of training volume, interval durations, and the balance between high- and low-intensity phases is also essential to refine HIIT’s application in hypertensive populations.

The present systematic review and meta-analysis provides important insights into the effects of high-intensity interval training (HIIT) on blood pressure (BP) in hypertensive individuals. Despite its strengths, this study and the included randomized clinical trials (RCTs) present several limitations: (i) The included RCTs exhibited substantial heterogeneity in HIIT protocols, with variations in intensity, frequency, and duration, which hinder standardization and reproducibility; (ii) most studies lacked long-term follow-up, limiting the ability to assess sustained blood pressure (BP) reductions or broader cardiovascular outcomes; (iii) there was an inconsistent use of intensity control methods, with some studies relying on subjective metrics like self-selected intensity (SSI) instead of objective measures such as %VO_2peak_ or %HR_max_. These inconsistencies may have contributed to the modest reductions observed, particularly in systolic BP (SBP); (iv) finally, the lack of ambulatory BP monitoring and the predominance of studies with small sample sizes and short intervention periods further restrict the generalizability and clinical applicability of the findings.

Future research should aim to standardize HIIT protocols, incorporating precise intensity control methods and tailoring interventions to individual patient characteristics such as age, sex, fitness level, and comorbidities. Long-term trials are essential to evaluate sustained BP reductions and adherence, while integrating ambulatory BP monitoring would provide insights into 24 h BP variability. Studies exploring endothelial function, autonomic regulation, and oxidative stress are needed to clarify the pathways underlying HIIT’s antihypertensive effects. By addressing these gaps, future research can strengthen the evidence base for HIIT as a time-efficient, non-pharmacological strategy in hypertension management.

## 5. Conclusions

This meta-analysis provides evidence that high-intensity interval training (HIIT) offers modest reductions in systolic blood pressure (SBP) among hypertensive individuals, with no significant impact on diastolic blood pressure (DBP). The observed SBP reduction, although statistically significant, falls short of clinical relevance when compared to pharmacological treatments and other non-pharmacological strategies such as sodium reduction.

These findings underscore the potential of HIIT as a supplementary, time-efficient intervention to support cardiovascular health and lifestyle modification in hypertensive populations. However, its efficacy as a standalone therapy remains limited, particularly in achieving clinically meaningful reductions in blood pressure.

The variability in HIIT protocols across the studies highlights the need for standardized intensity control and progression strategies to optimize outcomes. Future research should focus on refining HIIT prescription parameters, such as %HR_max_, %VO_2peak_, and training volume, to enhance its reproducibility and effectiveness. Furthermore, integrating HIIT with other exercise modalities or lifestyle interventions may amplify its benefits, offering clinicians and healthcare practitioners a broader spectrum of tools for hypertension management.

For health professionals, this study reinforces the importance of personalized exercise prescriptions, particularly when incorporating HIIT into treatment plans for hypertensive patients. Adherence to structured protocols and close monitoring of intensity levels are critical to maximizing the intervention’s benefits while ensuring patient safety.

## Figures and Tables

**Table 1:** Participants, intervention, comparators, outcomes, study design (PICOS) criteria for inclusion of randomized clinical trials.

Population	Hypertensive adults (≥18 Years)
Intervention	High-intensity interval training (HIIT) involving either cycling or treadmill
Comparison	Intervention group (HIIT) with another intervention group or a control group without exercise
Outcomes	Systolic and diastolic blood pressure
Study design	Human randomized controlled trials

**Table 2:** Characteristics of the RCTs included in the current systematic review and meta-analysis.

Study	Groups (Control/Intervention)		Sex	Mean Age	Duration	Type of HIIT	Exercise Intervention	SBP		DBP		Pharmacological Control	Intensity Control	Intensity Manipulation
Gorostegi et al. (2018) [62]	EG_1_: 44 EG_2_: 44	CG: 45	Mixed	54.0 ± 8.2	2 d/16 wk	Treadmill and Cycling	High-volume HIIT (4 × 4 min at R3 and 29 min at R2 of recovery) and low-volume HIIT (2 × 4 min at R3 and 12 min at R2 of recovery)	EG_1_: ↓ EG_2_: ↓	GC: ↓	EG_1_: ↓ EG_2_: ↓	GC: ↓	ACEIs ARBs Diuretics CCB β-B Statins	%VO_2peak_	As the weeks went by, the % of VO_2peak_ increased.
Izadi et al. (2018) [63]	EG: 15	CG: 15	Mixed	61.7 ± 5.7	3 d/6 wk	Cycling	1.5 min interval at 85–90% of heart rate reserve (HRR) and 2 min active phase at 50–55% of HRR	EG: ↓	CG: ↑	EG: ↓	CG: ↑	Yes but it does not specify	%HR_R_	NR
Lins-Filho et al. (2020) [64]	EG_1_: 20 EG_2_: 20 EG_3_: 20 EG_4_: 20	CG: 20	Female	65.3 ± 4.2	8 sessions	Treadmill	10 sets of 1 min at 20%/30%/40% more than the SSEIwith 1 min of recovery at 20%/30%/40% less than the SSEI, or 5 sets of 2 min 80–95% VO_2peak_ with 2 min of recovery at 40–50% VO_2peak_	EG_1_: ↔ EG_2_: ↔ EG_3_: ↔ EG_4_: ↔	CG: ↔	EG_1_: ↔ EG_2_: ↔ EG_3_: ↔ EG_4_: ↔	CG: ↔	NR	SSI and %VO_2peak_	Changed the degree and speed as the subject desired.
Martinez et al. (2020) [65]	EG_1_: 61 EG_2_: 62	CG: 59	Mixed	53.7 ± 8.0	2 d/16 wk	Treadmill and Cycling	High-volume HIIT (4 × 4 min at R3 and 29 min at R2 of recovery) and low-volume HIIT (2 × 4 min at R3 and 12 min at R2 of recovery)	EG_1_: ↓ EG_2_: ↓	CG: ↓	EG_1_: ↓ EG_2_: ↓	CG: ↓	ACEIs ARBs Diuretics CCB β-B Statins	Each participant’s HR	NR
Soltani et al. (2020) [66]	EG_1_: 10 EG_2_: 10	GC: 10	Male	47.9 ± 3.2	3 d/8 wk	Treadmill	4 or 27 repetitions of 30 s or 4 min activity at 85–90% of VO_2peak_ and active recovery at 15%–30% of VO_2peak_	EG_1_: ↓ EG_2_: ↓	CG: ↔	NR	NR	ARBs	%VO_2peak_	As the weeks went by, the % of VO_2peak_ increased.
Taha et al. (2023) [67]	EG:30	CG:30	Female	40.0 ± 10.0	3 d/12 wk	Cycling	4 min of cycling at 85–90% of HR_max_ interspersed with 3-min active recovery time at 60–70% of HR_max_.	EG: ↓	CG: ↓	EG: ↓	CG: ↓	Yes but it does not specify	%HR_max_	As the weeks went by, the % of HR_max_ increased.
Twerenbold et al. (2023) [68]	EG: 19	CG: 19	Mixed	58.0 ± 7.0	3 d/8 wk	Treadmill	4 × 4 min of high-intensity intervals at an intensity equivalent 80–95% HR_max_ with active recovery of 3 min.	EG: ↔	CG: ↔	EG: ↔	CG: ↔	CCB β-B	% HR_max_	NR

Abbreviations: CG: control group, d: day, EG: experimental group, HIIT: high-intensity interval training, R2 and R3: Ventilatory Thresholds, Skinner’s Three-Phase Model, HR: heart rate, HR_max_: maximum heart rate, HRR: heart rate reserve, min: minutes, NR: non-report, s: seconds, SSI: self-selected intensity, SSEI: self-selected exercise intensity, SSP: self-selected pace, VO_2peak_: peak oxygen consumption, wk: week, ACEIs: angiotensin-converting enzyme inhibitors, ARBs: angiotensin II receptor blockers, CCB: calcium channel blocker, β-B: beta-blockers.

## Data Availability

The original contributions presented in the study are included in the article; further inquiries can be directed to the corresponding authors.
